# Comparative Digital Gene Expression Analysis of the *Arabidopsis* Response to Volatiles Emitted by *Bacillus amyloliquefaciens*

**DOI:** 10.1371/journal.pone.0158621

**Published:** 2016-08-11

**Authors:** Hai-Ting Hao, Xia Zhao, Qian-Han Shang, Yun Wang, Zhi-Hong Guo, Yu-Bao Zhang, Zhong-Kui Xie, Ruo-Yu Wang

**Affiliations:** 1 Gaolan Station of Agricultural and Ecological Experiment, Cold and Arid Regions Environmental and Engineering Research Institute, Chinese Academy of Sciences, Lanzhou, China; 2 Key Laboratory of Stress Physiology and Ecology in Cold and Arid Regions of Gansu Province, Lanzhou, China; 3 Key Laboratory of Desert and Desertification, Cold and Arid Regions Environmental and Engineering Research Institute, Chinese Academy of Sciences, Lanzhou, China; 4 University of Chinese Academy of Sciences, Beijing, China; KRIBB, REPUBLIC OF KOREA

## Abstract

Some plant growth-promoting rhizobacteria (PGPR) regulated plant growth and elicited plant basal immunity by volatiles. The response mechanism to the Bacillus amyloliquefaciens volatiles in plant has not been well studied. We conducted global gene expression profiling in *Arabidopsis* after treatment with *Bacillus amyloliquefaciens* FZB42 volatiles by Illumina Digital Gene Expression (DGE) profiling of different growth stages (seedling and mature) and tissues (leaves and roots). Compared with the control, 1,507 and 820 differentially expressed genes (DEGs) were identified in leaves and roots at the seedling stage, respectively, while 1,512 and 367 DEGs were identified in leaves and roots at the mature stage. Seventeen genes with different regulatory patterns were validated using quantitative RT-PCR. Numerous DEGs were enriched for plant hormones, cell wall modifications, and protection against stress situations, which suggests that volatiles have effects on plant growth and immunity. Moreover, analyzes of transcriptome difference in tissues and growth stage using DGE profiling showed that the plant response might be tissue-specific and/or growth stage-specific. Thus, genes encoding flavonoid biosynthesis were downregulated in leaves and upregulated in roots, thereby indicating tissue-specific responses to volatiles. Genes related to photosynthesis were downregulated at the seedling stage and upregulated at the mature stage, respectively, thereby suggesting growth period-specific responses. In addition, the emission of bacterial volatiles significantly induced killing of cells of other organism pathway with up-regulated genes in leaves and the other three pathways (defense response to nematode, cell morphogenesis involved in differentiation and trichoblast differentiation) with up-regulated genes were significantly enriched in roots. Interestingly, some important alterations in the expression of growth-related genes, metabolic pathways, defense response to biotic stress and hormone-related genes were firstly founded response to FZB42 volatiles.

## Introduction

Plant growth-promoting rhizobacteria (PGPR) comprise a wide range of bacteria that colonize roots, which have beneficial effects in enhancing plant productivity and they often elicit plant immunity against multiple plant pathogens in various plant species [1–3]. At the same time, the recent studies have also demonstrated that PGPR can promote plant growth and elicit disease resistance without physical contact by releasing volatiles [4–10]. Ryu et al. first reported the promotion of plant growth by bacterial volatiles [4] and many subsequent studies have demonstrated the effects of bacterial volatiles on the growth of plants [4, 5, 11–20]. Moreover, extensive studies have identified the mechanism of plant response to bacterial volatiles [12, 21]. However, the present knowledge in completely understanding the response mechanism of plant elicited by bacteria volatiles is not sufficient, such as information on the different growth stages and/or tissues of host plant.

The NGS technologies have emerged to offer an opportunity to exhaustively sample transcripts and digitally measure transcription levels in all organisms including Arabidopsis thaliana with an important model plant for studying the plant response mechanism under different treatments or conditions [22, 23]. DGE is a tag-based transcriptome sequencing tool in the analysis of gene expression when comparing two very similar samples [24, 25]. The advantage of DGE is sensitive and cost-effective the larger dynamic range obtained per experiment [26–28].

*B*. *amyloliquefaciens* FZB42 is a Gram-positive PGPR and it was first isolated from sugar beet in Brandenburg, Germany [29]. In 2007, the complete genome sequence of FZB42 was published and unexpectedly, it was shown to have the potential to produce abundant secondary metabolites [30]. Further research have demonstrated that the secondary metabolites of FZB42 include antifungal, antibacterial, and nematocidal components, such as lipopeptides and polyketides [31]. Idris et al. reported the plant growth promotion potential of FZB42 according to the Trp-dependent synthesis of auxins [32]. Subsequently, the study identified the successful colonization ability in different plants and the positive response to plant root exudates [33]. So, FZB42 has become a popular study strain because of its potential capacity for biocontrol and plant growth promotion, [34, 35].

In the present study, we performed the first digital gene expression (DGE) analysis of *A*. *thaliana* when exposed to volatiles emitted by *B*. *amyloliquefaciens* using the Illumina sequencing technique. We determined the expression profile of *A*. *thaliana* for the major metabolic pathways as well as detecting candidate genes affected by the volatiles released by *B*. *amyloliquefaciens*. This is the first transcriptomic study to identify differentially expressed genes (DEGs) and pathways in different tissues and growth stages in *A*. *thaliana* when exposed to *B*. *amyloliquefaciens*.

## Materials and Methods

### Preparation of materials and treatments

*A*. *thaliana* (Col-0) seeds were surface-sterilized (soaking in 70% ethanol for 2 min, followed by soaking in 1% sodium hypochlorite for 10 min), rinsed four times with sterile distilled water, and then planted in a 500 mL bottle that contained a small bottle (10 mL), before vernalizing for 2 days at 4°C in the absence of light and placing in the growth room. Both bottles contained Murashige and Skoog salt (MS) medium adjusted to pH 5.7, containing 1.5% sucrose and 0.8% agar, which was also used to fix the small bottle with solidified agar. The temperature was maintained at 221°C and the growth room had a light: dark cycle of 16:8 h. Each large bottle contained 40 mL of MS and the small bottle contained 4 mL MS.

For the experiments, *B*. *amyloliquefaciens* FZB42 was grown overnight in 20 mL test tubes in Luria-Bertani (LB) broth and then diluted with LB broth to OD = 0.050. Next, 50 μL of the culture or water (control) was applied to the small bottle in the larger bottle, which contained five plants. The cultures and seeds were added to the small bottle and larger bottle at the same time, thereby allowing a dynamic relationship.

At 23 days after inoculation, we observed about 10 rosette leaves, which differed significantly in the *Arabidopsis* plant treated with FZB42 because they were larger than the control (water-treated) plants. This stage was defined as the “seedling period.” At 71 days after inoculation, no significant difference was observed because the plants almost filled the whole bottles. This stage was defined as the “mature period” when the first bud appeared.

### Plant biomass Measurements

#### Fresh and dry weight

Each treatment has at least three repetitions. Samples of each replication (n ≥ 4) were fresh weighed. And fresh sample material will be drying for 30 min at 105°C oven, turn to 60°C drying to constant weight, then weighed dry weight.

#### Chlorophyll measurement

Two and three weeks after bacterial treatment, Arabidopsis leaves were detached (one leaf per plant, four plants total) and chlorophyll amounts were determined spectrophotometrically. Approximately 0.02 g tissue was ground with 2 mL 80% acetone (20% water) in Scale test tubes (Fisher Scientific), followed by digestion at 48 h at room temperature, until the leaves turned white. 1ml the extracting solution was mixed into 1ml 80% acetone, the final liquids was used spectrometric measurements at wavelengths of 646.8 and 663.2 nm. Total chlorophyll content was calculated using the formula as reported by Lichtentthaler25: total chlorophyll = (7.15 * A663.2 + 18.71 * A646.8)/1000/ (fresh weight of leaves); calculate values were reported as g Chl per g FW (n = 4).

### RNA isolation and quantification

For“seedling period”, the leaves and roots treated with FZB42 volatiles (E03: Leaves-volatiles treated; E04 Roots- volatiles treated), or water as the control (E01: Leaves Control; E02: Roots Control), were harvested after treatment. Similarly, for “mature period”, the leaves and roots treated with FZB42 volatiles (E07: Leaves-volatiles treated; E08: Roots- volatiles treated), or water as the control (E05: Leaves Control; E06: Roots Control), were harvested at the mature stage after treatment, and they were then prepared to extract RNA. We consider the seedling stage as an example because the whole process was the same for each stage. For each stage, one sequencing sample comprised three biological replicates (three bottles). Each replicate comprised five seedlings (a bottle), where the plants were transferred rapidly to a mortar filled with liquid nitrogen and the leaves and roots were carefully separated immediately using tweezers while the plant was in a brittle state. Total RNA was extracted from the separated tissues at each stage using RNAiso Plus (Takara), according to the manufacturer’s instructions. The isolated total RNA was separated on 1% agarose gels. The purity of RNA (OD260/ 280) was determined with a NanoDrop 2000^™^ spectrophotometer (Thermo Scientific, Waltham, MA, USA). We submitted the final RNA samples to Biomarker Technologies Co. Ltd (http://www.biomarker.com.cn) for further quality control analysis. The final high-quality RNA was used for DGE library preparation, sequencing, and bioinformatics analysis, which was performed by the same company and further confirmed using a 2100 Bioanalyzer (Agilent Technologies).

### DGE library preparation and sequencing

#### DGE library construction

After confirming their quality, the samples were employed to construct a library, where the main process used an Illumina DGE tag profiling kit according to the manufacturer’s protocol, as follows: (1) mRNA was captured with Oligo (dT) mRNA magnetic beads; (2) mRNA was broken into short segments by adding fragmentation buffer; (3) using the mRNA as a template, the first strand cDNA was synthesized by six random hexamers, and the second strand and cDNA was then synthesized by adding buffer, dNTPs, RNaseH, and DNA polymerase I; (4) the double-stranded cDNA was purified with AMPure XP beads, repaired, A-tails were added, and the joints were sequenced; (5) fragment size selection was performed using AMPure XP beads and the cDNA library was obtained by PCR enrichment.

#### Quality control for the library

After building the library, preliminary quantitative analysis was performed using Qubit2.0, where the insert size in the library was detected using an Agilent 2100 Bioanalyzer. The effective concentration of the library was detected using quantitative PCR (qPCR) to ensure the quality of the library.

#### Sequencing

After determining the effective concentration and data quantification, the different libraries were pooled and sequenced by HiSeq2500. The read length employed for sequencing was SE50. The sequence data are available at the NCBI SRA under accession number SRP069127.

### Bioinformatics analysis

The bioinformatics analyses of the gene expression profiles were performed as follows.

#### Quality control

The original image files were translated into raw data (raw reads) in the fastq form by “base calling.” Clean data (clean reads) were obtained by removing the reads containing adapters and sequencing primers from the raw data. The base number, Q30, and GC contents of the clean data were calculated. All of the downstream analyses were based on the high-quality clean data.

#### Read mapping to the reference genome

Reference annotation files were downloaded directly from the genome databases website (//ftp.ensemblgenomes.org/pub/plants/release-25/fasta/arabidopsis_thaliana/TAIR10). All of the clean tags were mapped to the *Arabidopsis* reference sequence using TopHat2. We selected TopHat (http://ccb.jhu.edu/software/tophat/index.shtml) as the mapping tool because it [36] can recognize splice junctions based on the alignment results, thereby improving the overall utilization of the sequencing data.

#### Quantification of gene expression levels

We employed fragments per kilobase of transcript per million fragments mapped (FPKM) as the index to determine the transcript or gene expression levels using the Cufflinks (http://cufflinks.cbcb.umd.edu/) [37] tool based on a mathematical model, where the values ranged from10^−2^ to 10^4^ [38].

#### Differential expression analysis and DEG clustering analysis

In order to screen for DEGs, we used EBSeq (https://www.biostat.wisc.edu/~kendzior/EBSEQ/) [39] to analyze differential gene expression under two conditions. The Benjamini-Hochberg method was using to adjust the *P*-values in order to reduce the overall false positive rate. We employed the corrected *P*-value, false discovery rate (FDR) < 0.01, and fold change (FC) ≥ 2 as selection criteria, where FC represents the relative ratio of the expression amount between the two samples (conditions).

#### GO and Kyoto Encyclopedia of Genes and Genomes (KEGG) enrichment analysis of DEGs

Annotation information for the DEGs was obtained using BLAST (http://blast.ncbi.nlm.nih.gov/Blast.cgi) [40] based on the GO [41] and KEGG [42] databases. GO terms were considered significantly enriched for DEGs when the corrected *P*-value was less than 0.05. KEGG is a large-scale molecular dataset, which can facilitate the assignment of genes to pathways in order to further interpret the functions of genes (http://www.genome.jp/kegg/).

### Quantitative RT-PCR (qRT-PCR) validation

qRT- PCR was also performed to validate the expression of 17 representative DEGs identified by DGE profiling. Gene-specific primers were designed for each DEG according to the cDNAs (S1 Table) and the *Arabidopsis* actin12 gene AT3G46520 was used as an internal control [43]. Eight remaining RNA samples for DGE analysis were subjected to qRT-PCR analysis. qRT- PCR was performed using a TaKaRa SYBR Premix ExTaq II reagent kit. The relative quantitative method (^ΔΔ^CT) was used to calculate the FCs in the expression levels of target genes [44].

## Results

### Plant growth promotion with sustained FZB42 exposure

**Fig 1:**
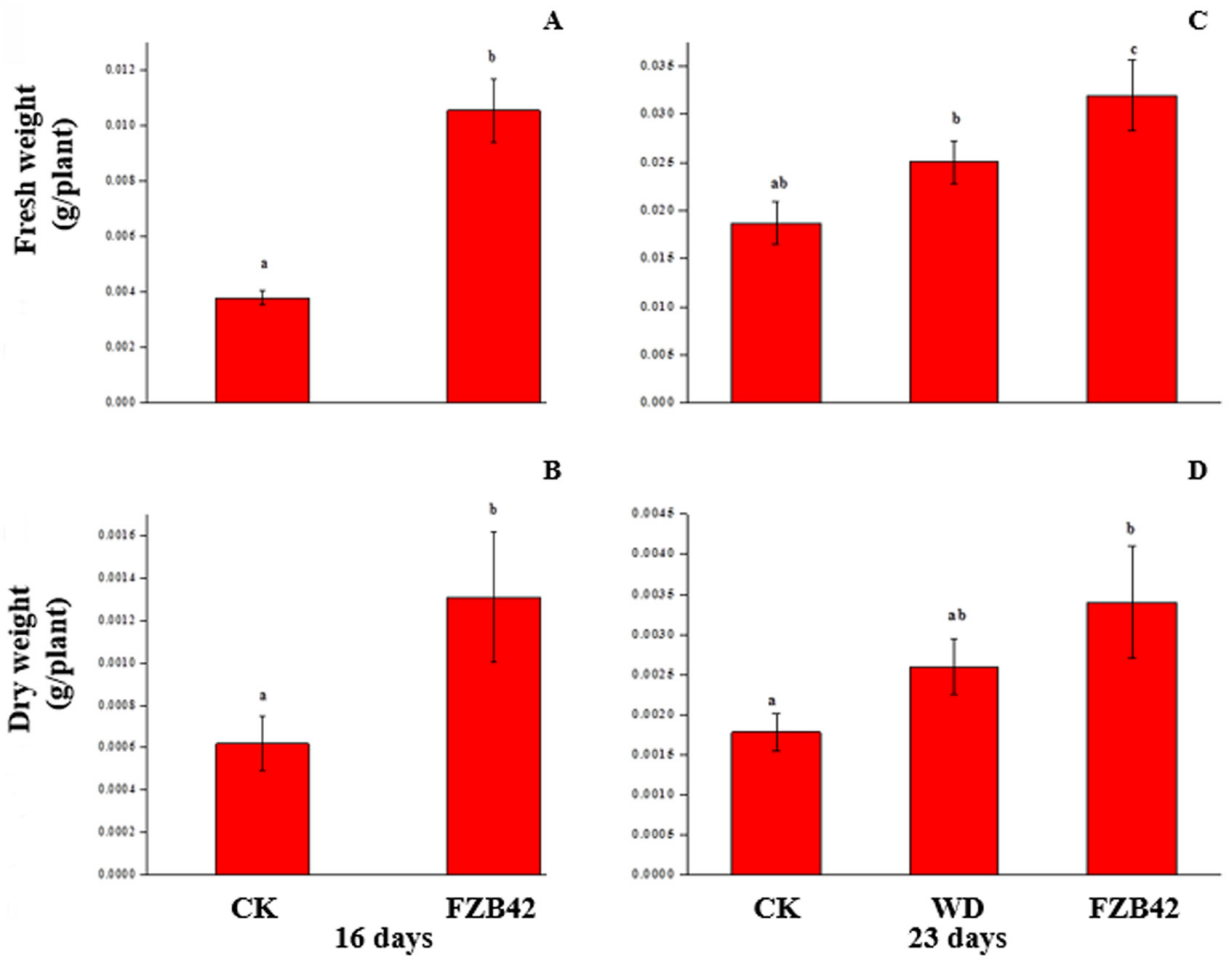
A, B, C and D. An increase in plant growth with sustained *Bacillus amyloliqefaciens* FZB42 exposure compared with FZB42 withdrawn at 16 day and water-treated controls as determined by fresh weight and dry weight. Fresh weight [A], and dry weight [B] for 16-days plants continuous exposed to FZB42 volatiles (n≥10) or water controls (n ≥10). Fresh weight [C], and dry weight [D] for 23-days old plants continuous exposed to FZB42 volatiles (n ≥10), FZB42 withdrawn at 16 day (n ≥10) and water controls (n ≥10). Different letters indicate significant differences between treatments (ANOVA, p ≤ 0.05).

**Fig 2:**
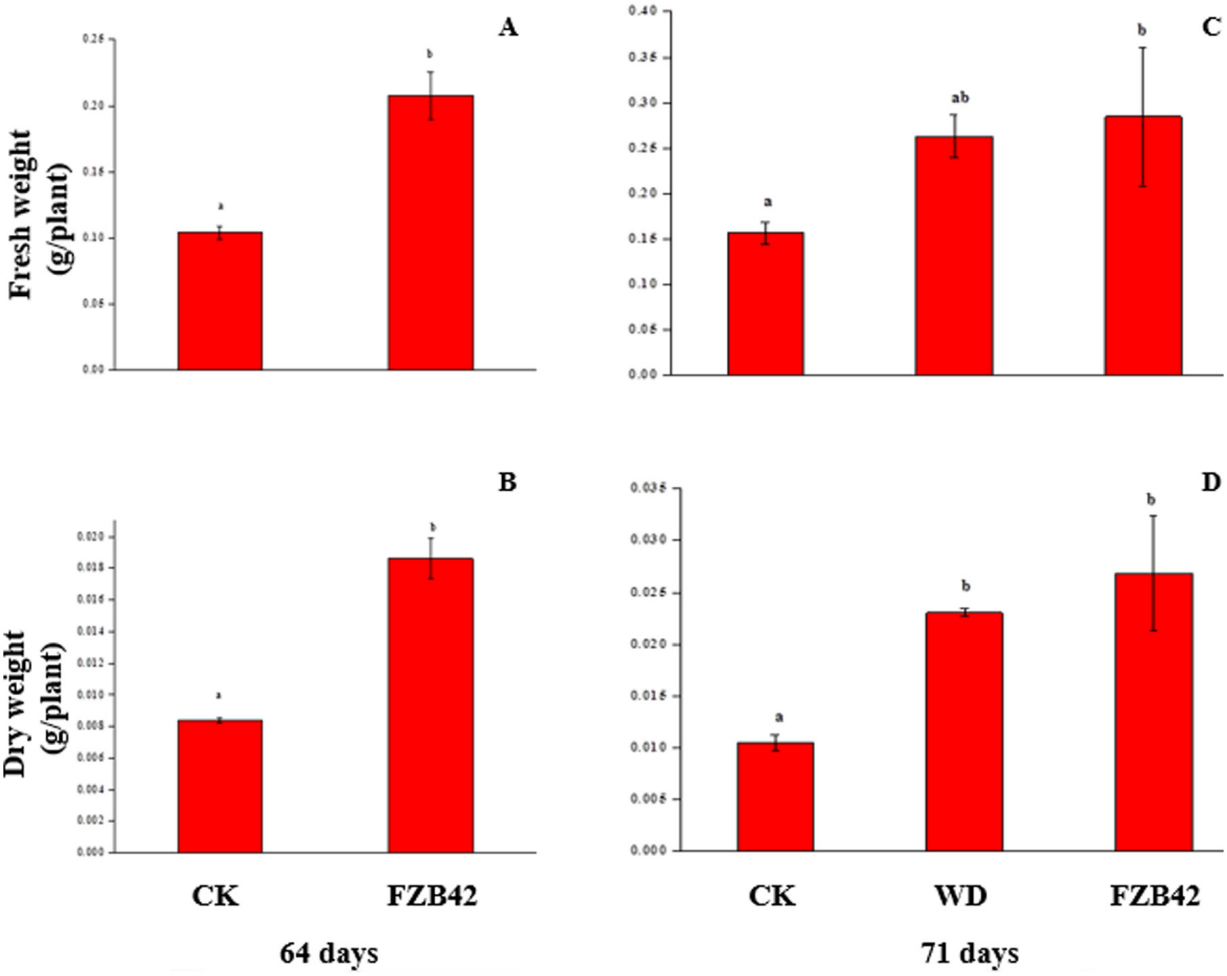
A, B, C and D. An increase in plant growth with sustained *Bacillus amyloliqefaciens* FZB42 exposure compared with FZB42 withdrawn at 64 day and water-treated controls as determined by fresh weight and dry weight. Fresh weight [A], and dry weight [B] for 65-days plants continuous exposed to FZB42 volatiles (n≥10) or water controls (n ≥10). Fresh weight [C], and dry weight [D] for 71-days old plants continuous exposed to FZB42 volatiles (n ≥10), FZB42 withdrawn at 65 day (n ≥10) and water controls (n ≥10). Different letters indicate significant differences between treatments (ANOVA, p ≤ 0.05).

To examine how long-term FZB42 exposure affects plant growth, Arabidopsis plants were treated with FZB42 volatiles during the full life cycle of the plant, which were grown in double-sized Magenta boxes containing half-strength MS media with 1.5% sucrose. After 16 and 64 days of FZB42 exposure plants exhibited a similar increase in fresh and dry weight respectively compared to water controls (Figs 1A, 1B, 2A and 2B **p < 0.05**).

**Fig 3:**
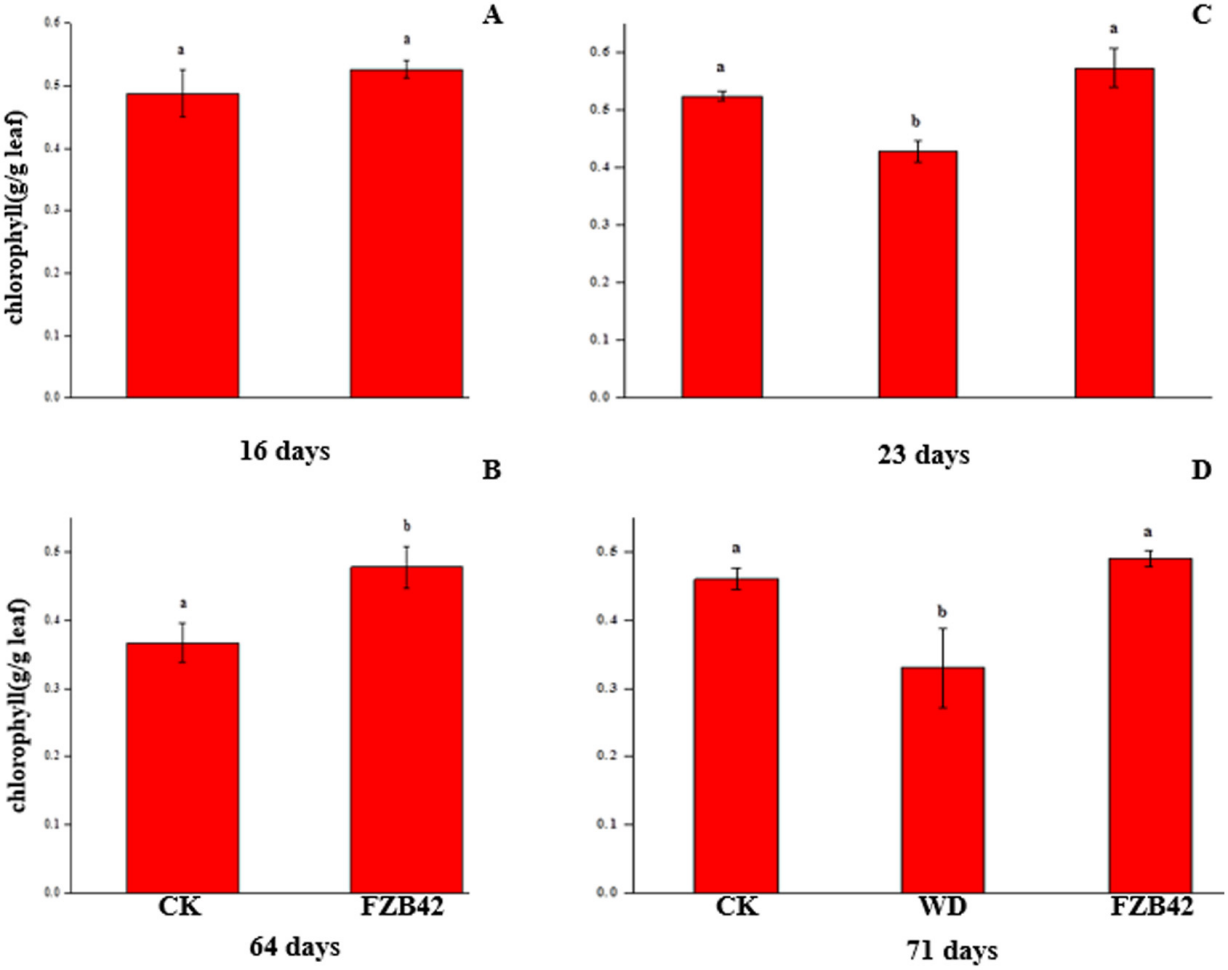
A, B, C and D. An increase in chlorophyll content with sustained FZB42 exposure compared with FZB42 withdrawn at 16, 65 day and water-treated controls. Chlorophyll content (Chl. a + b) (A, B, C and D), (A–D, n = 4). Different letters indicate significant differences between treatments (ANOVA, p ≤ 0.05).

To probe whether growth promotion requires a sustained or transient FZB42 signal, FZB42 cultures contained in vials were or were not withdrawn (WD) after 16 and 64 days from glass spawn bottle and growth measurements were taken at 23 and 71 days, respectively. An FZB42 exposure-dependent growth promotion was observed with fresh and dry weight with a very similar profile (Figs 1C, 1D, 2C and 2D). However, compared with water treatment alone, plant fresh and dry weight with 23-day FZB42 exposure was greater (Figs 1C and 2C **p < 0.05**), whereas fresh and dry weight with 16-day exposure was similar degree (Figs 1C and 2C **p > 0.05**). At mature stage, although the fresh and dry weight with 71-day FZB42 exposure increased than with the control (Figs 1D and 2D **p < 0.05**), the dry weight with 64-day FZB42 exposure was greater than the control (Figs 1D and 2D **p < 0.05**). Beside, 16 and 64 day FZB42-exposure increased total chlorophyll (Chl. a+b) content, but the total chlorophyll (Chl. a+b) with 64- day FZB42 exposure was significantly increased than the control (Fig 3A and 3B, **p < 0.05**). Sustained FZB42 exposure preserved elevated chlorophyll levels at 23 and 71 day than water controls, but which was not significantly different from water controls (p > 0.05) (Fig 3C and 3D, **p < 0.05**). In contrast, an abbreviated 16 and 64-day FZB42 exposure total chlorophyll were greater than the water control.

### DGE library sequencing

As shown in Table 1, eight DGE libraries were obtained from the roots and leaves of the control- and *B*. *amyloliquefaciens* FZB42 volatiles-treated seedling and mature *Arabidopsis* plants using high-throughput sequencing. We generated approximately 11 million raw tags for each library. The total number of filtered clean reads generated varied from 10.06 to 12.05 million and the number of mapped reads ranged from about 9.4 to 11.47 million per library, which represented more than 94% of the clean reads based on the *Arabidopsis* genome (TAIR10). The distributions of the total and distinct clean tags in the eight libraries are shown in S1 File. The results demonstrate that the distributions of the tag expression patterns were similar for each library.

**Table 1:** Categorization and abundances of DGE tags.

Summary		E01	E02	E03	E04	E05	E06	E07	E08
Raw Data	Total	10712865	10126012	10082118	10402577	11607059	12635834	12443065	12388226
Raw Data	Distinct Tag	6161655	6423192	6316849	6635083	7225031	7870495	7063367	6929547
Clean tag	Total number	10690006	10089026	10063386	10388538	11072798	12050981	11852073	10503468
Clean tag	Distinct Tag number	6156026	6417135	6312695	6629921	6740432	7341852	6528554	6300500
Tag Mapping to Gene	Total number	7790350	7214870	7416280	7507460	9732080	10803400	10358000	8499460
Unambiguous Tag Mapping to Gene	Total number	7790350	7214870	7416280	7507460	9732080	1080340	10358000	8499460
Unambiguous Tag Mapping to Gene	Total % of clean tag	0.728751	0.715121	0.736957	0.722668	0.878918	0.896475	0.87394	0.809205
Tag-mapped Genes	number	20755	21336	20804	21611	21084	21908	21509	21826
Tag-mapped Genes	% of ref genes	0.034635	0.035605	0.034717	0.036064	0.035184	0.036559	0.035893	0.036422
Unambiguous Tag-mapped Genes	number	20755	21336	20804	21611	21084	21908	21509	21826
Unambiguous Tag-mapped Genes	% of ref genes	0.034635	0.035605	0.034717	0.036064	0.035184	0.036559	0.035893	0.036422
Mapping to Genome	Total number	2194185	2194277	1987655	2157144	826525	665368	964545	1414876
Mapping to Genome	Total % of clean tag	0.205256	0.217491	0.197514	0.207647	0.074645	0.055213	0.081382	0.134706
Mapping to Genome	Distinct Tag number	5155470	5414267	5329173	5597165	5893825	6373233	5690190	5444582
Unknown Tag	Total number	705471	679879	659451	723934	514193	582213	529528	589132
Unknown Tag	Total % of clean tag	0.065994	0.067388	0.06553	0.069686	0.046437	0.048312	0.044678	0.056089
Unknown Tag	Distinct Tag number	668983	642224	6312695	6629921	496366	555608	504624	546701
Unknown Tag	Distinct Tag % of clean tag	0.06258	0.063656	0.627293	0.638196	0.044828	0.046105	0.042577	0.05205

Clean tags represent the tags that remained after filtering low-quality tags from the raw data. Distinct tags are different tags and unambiguous tags are the remaining clean tags after removing tags mapped to more than one locus in the reference genome.

### Analysis of DEGs

**Fig 4:**
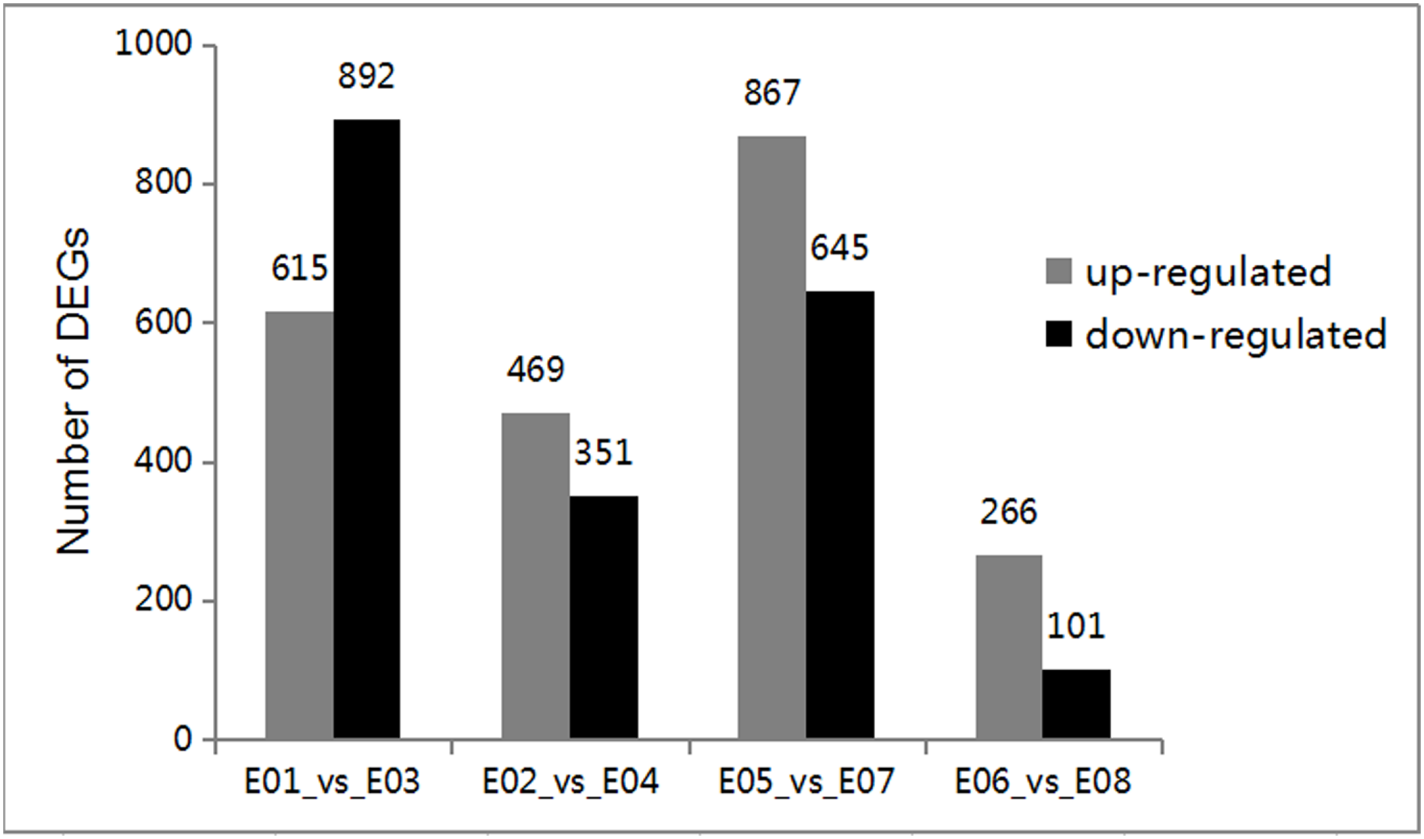
Number of differentially expressed genes in each comparison. The numbers of up-regulated (in red) and down-regulated genes (in blue) are presented.

To analyze the DEGs, FDR ≤ 0.01 and |log2Ratio| ≥ 2 were used as the threshold to define the significance of differential gene expression. In total, 367, 820, 1507, and 1512 DEGs were detected in E06 vs. E08, E02 vs. E04, E01 vs. E03, and E05 vs. E07, respectively (S2 Table). The results showed that 615 DEGs were significantly upregulated and 892 DEGs were downregulated in the E03 library compared with the E01 library (Fig 4). By contrast, 469 DEGs were significantly upregulated and 351 DEGs were downregulated in E04 compared with E02. Furthermore, 867 DEGs were upregulated and 645 genes were downregulated in E07 compared with E05. The lowest number of DEGs was found in E08 compared with E06. In addition, 266 DEGs were upregulated and 101 genes were downregulated in E08 compared with E06. As shown in Fig 4, more DEGs were upregulated in roots (E02 vs. E04) compared with leaves (E01 vs. E03), thereby suggesting that some genes were upregulated in the roots at the seedling stage in response to volatiles. The expression patterns were different in the leaves and roots at the mature stage.

**Fig 5:**
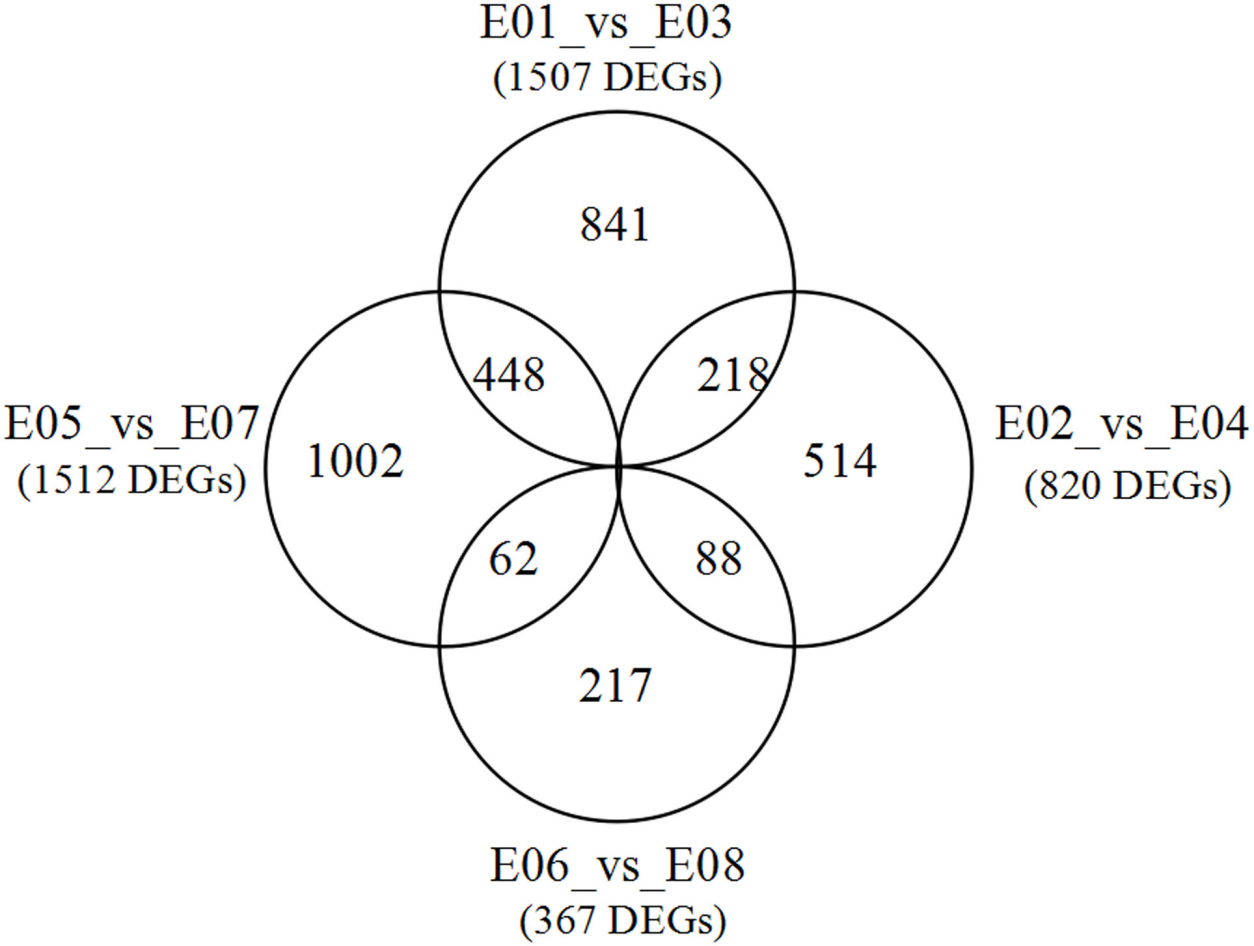
Venn diagram of genes differentially expressed in pair comparison of each library.

Fig 5 shows the distributions of DEGs in leaves and roots at seedling and mature stages. At seedling stage, 218 DEGs were shared in leaves and roots. But, only 62 DEGs were shared in these two tissues at mature stage. In contrast, total 448 DEGs were shared in leaves at two growth stages. For roots, the two growth stages shared 88 DEGs.

### Functional annotation of DEGs

**Fig 6:**
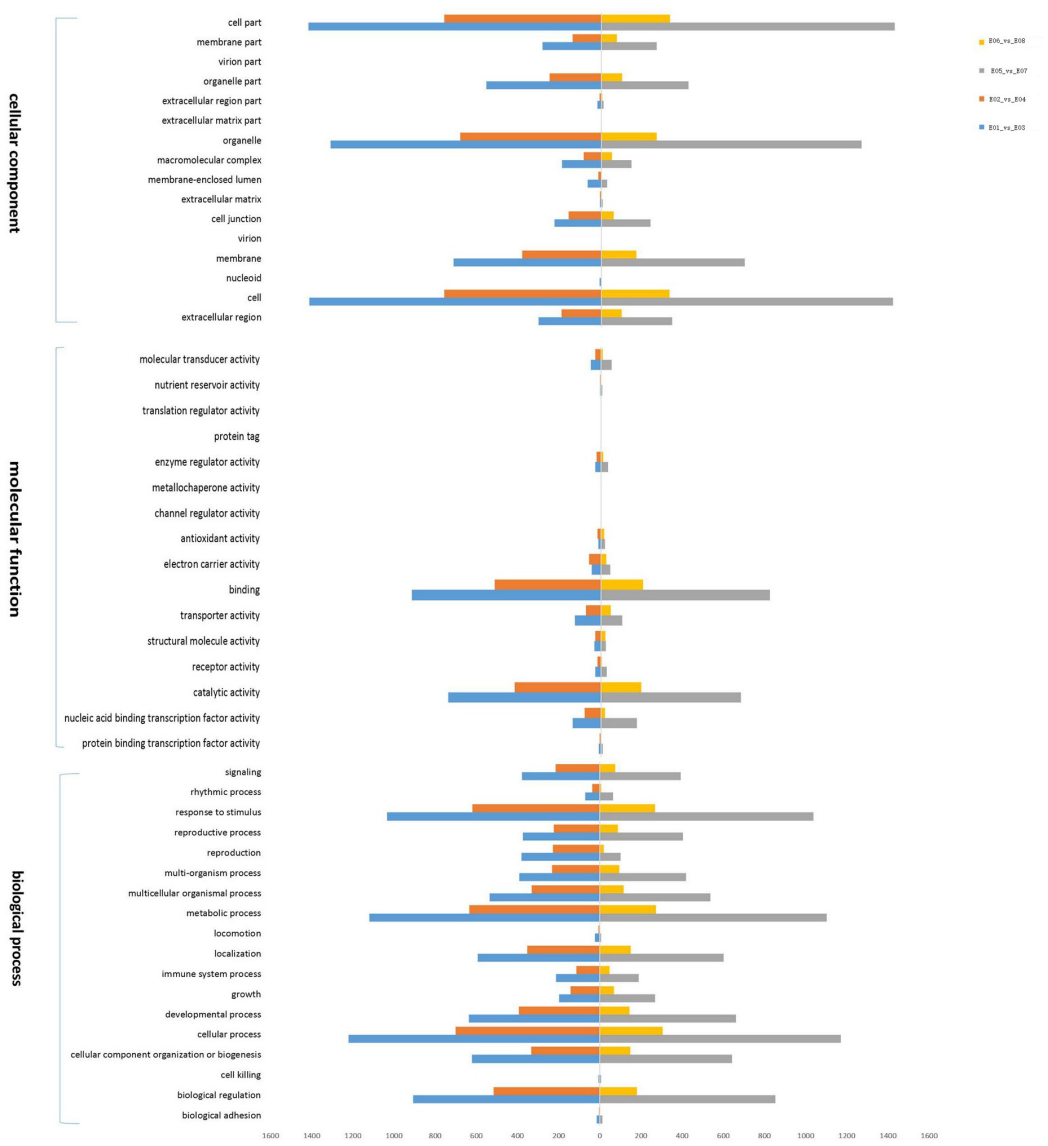
Gene Ontology (GO) functional annotation of differentially expressed genes based on RNA-Seq data in E01 vs E03 (blue), E02 vs E04 (dark red), E05 vs E07 (purple) and E06 vs E08 (yellow). The three main categories (biological process, cellular component, and molecular function) were used for GO analysis.

The functional categories of the DEGs in each group comparison were annotated with GO terms, including Cellular Component, Molecular Function, and Biological Process (Fig 6). As shown in Fig 6, the first three categories of the secondary annotation results for Cellular Component were cell, cell part, and organelle, while binding and catalytic activity were the main annotations represented in the Molecular Function category (Fig 6). For the Biological Process category, DEGs involved in responses to stimulus, metabolic process, cellular process, and biological regulation were strongly represented in E01 vs. E03, E02 vs. E04, E05 vs. E07 and E06 vs. E08 (Fig 6).

**Fig 7:**
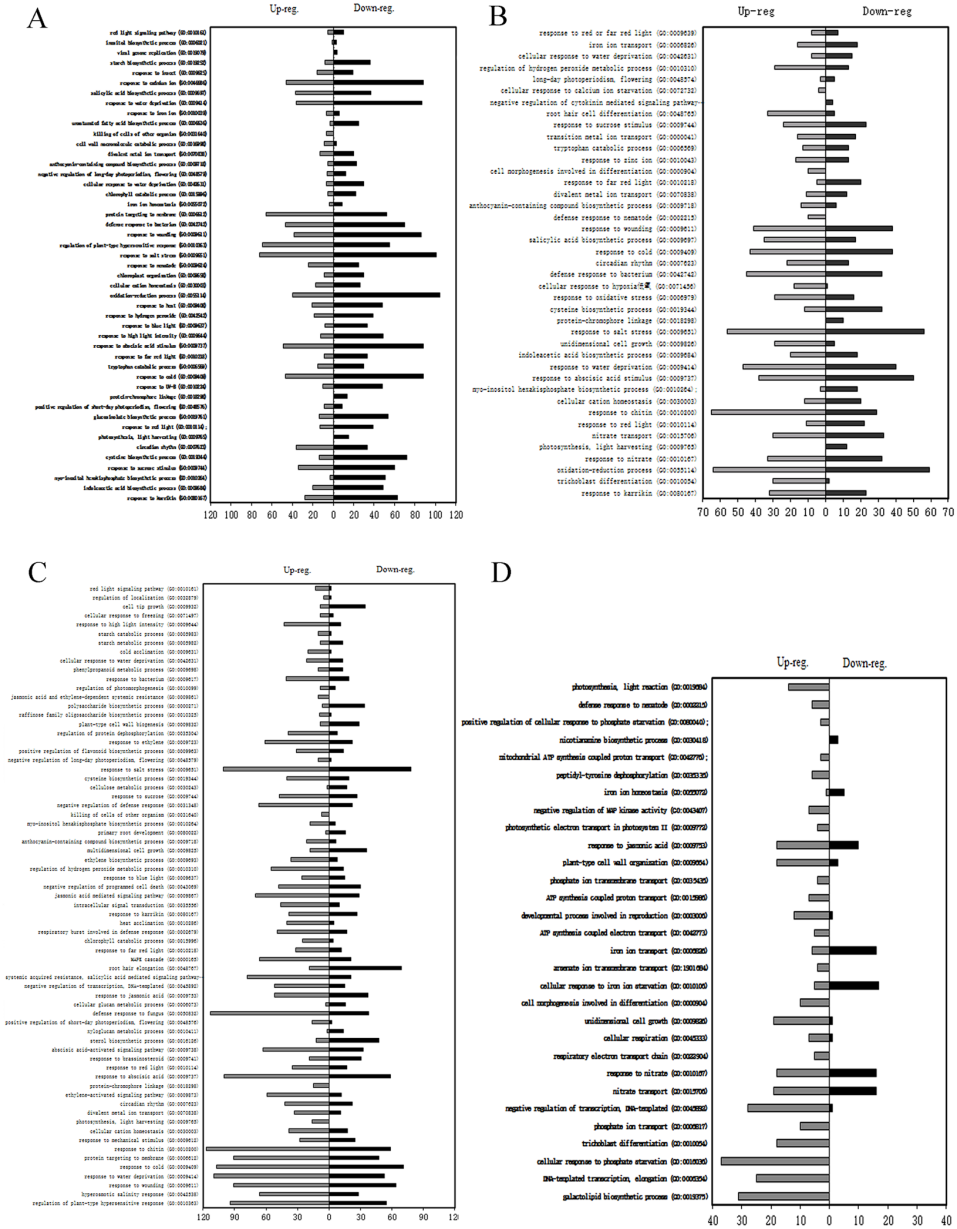
GO annotation of DEGs assigned to the biological process (BP) category after FZB42 volatiles triggered at seedling and mature stages. Panel A and B represent the DEGs in leaf and root tissues at seedling stage, respectively. And panel C and D represent the DEGs in leaf and root tissues at mature stage, respectively. GO enrichment analysis was conducted using topGO tool and GO terms with a p < 0.05 were considered to have significantly enriched expression in the cluster.

To further analysis significantly enriched GO terms in the biological process category using topGO software with Q values ≤ 0.05 (red border region). In leaves (Fig 7A and 7B), the two Go terms photosynthesis, light harvesting (GO:0009765) and protein- chromophore linkage (GO:0018298) were significantly enriched in both growth stages (seedling and mature), but DEGs annotated these two terms were down regulated at seedling stage and up regulated at mature stage. We found the term killing of cells of other organism (GO:0031640) with upregulated genes were significantly enriched in both growth stages (seedling and mature). GO:0019079 (Viral genome replication) with down-regulated genes was significantly enriched at seedling stage but not mature stage. However, GO:0009861 (jasmonic acid and ethylene-dependent systemic resistance) with up-regulated genes was an exception at mature stage, in which it was significantly enriched compared with at seedling stage.

In roots (Fig 7C and 7D), defense response to nematode (GO:0002215), cell morphogenesis involved in differentiation (GO:0000904) and trichoblast differentiation (GO:0010054) with upregulated genes were significantly enriched in roots at both seedling and mature stages. protein-chromophore linkage (GO:0018298), photosynthesis, light harvesting (GO:0009765) and negative regulation of cytokinin mediated signaling pathway (GO:0080037) with down- regulated genes were significantly enriched in roots at seedling stage. Only cellular response to calcium ion starvation (GO:0072732) was strongly induced among the upregulated genes in roots at seedling stage. Fifty of pathways with up-regulated genes were significantly induced in roots at mature stage including photosynthesis, light reaction (GO:0019684), photosynthetic electron transport in photosystem II (GO:0009772), negative regulation of MAP kinase activity (GO:0043407) and so on.

Besides, the other plant hormone-related pathways were also induced in leaves and/or roots. Salicylic acid biosynthetic process (GO: 0009697), indoleacetic acid biosynthetic process (GO: 0009684), and response to abscisic acid stimulus (GO: 0009737) were found at seedling stage. Response to abscisic acid stimulus (GO: 0009737), abscisic acid-activated signaling pathway (GO: 0009738), ethylene-activated signaling pathway (GO: 0009873), ethylene biosynthetic process (GO: 0009693), response to ethylene (GO: 0009723), response to jasmonic acid (GO: 0009753), jasmonic acid mediated signaling pathway (GO: 0009867) and response to brassinosteroid (GO: 0009741) were induced in leaves at mature stage. Response to jasmonic acid (GO: 0009753) was induced in roots at mature stage.

We also performed biochemical pathways analysis using the KEGG Pathway database (Table 2 and S2 File). In total, we assigned 295, 143, 211 and 91 DEGs to 94, 72, 74 and 47 KEGG pathways in E01 vs. E03, E02 vs. E04, E05 vs. E07 and E06 vs. E08, respectively. For significantly enriched pathways with Q values ≤ 0.05 in the KEGG database (red border region), we found that three, four, five and six KEGG pathways were significantly enriched in E05 vs. E07, E06 vs. E08, E01 vs. E03 and E02 vs. E04, respectively. Photosynthesis pathway was induced in leaves and roots at two growth stages. Interestingly, we found the expression of photosynthesis related genes was down regulated at seedling stage and up regulated at mature stage. Glucosinolate biosynthesis pathway was induced only in leaves at seedling stage and the genes related to glucosinolate biosynthesis pathway were all down regulated. Moreover, phenylalanine metabolism and phenylpropanoid biosynthesis were induced in roots at two growth stages and the number of genes with up- regulated was higher than the number of genes with down-regulated (S2 File).

**Table 2:** KEGG Pathway Enrichment Analysis of DEGs.

Pathway	DEGs with pathway annotation (295)	Corr_p_value	Pathway ID
**E01vsE03**			
photosynthesis-antenna proteins	17(5.76%)	3.9696E-13	ko00196
Glucosinolate biosynthesis	12(4.07%)	9.851E-09	ko00966
Circadian rhythm-plant	10(3.39%)	0.0055894	ko04712
Photosynthesis	17(5.76%)	0.011105	ko00195
Porphyrin and chlorophyll metabolism	11(3.73%)	0.024087	ko00860
**E02vsE04**			
Photosynthesis-antenna proteins	12(8.39%)	3.38E-10	ko00196
Phenylalanine metabolism	13(9.09%)	4.46E-03	ko00360
Stilbenoid,diarylheptanoid and gingerol biosynthesis	11(7.69%)	4.95E-03	ko00945
Limonene and pinene degradation	11(7.69%)	7.40E-03	ko00903
Circadian rhythm-plant	7(4.90%)	7.44E-03	ko04712
Phenypropanoid biosynthesis	13(9.09%)	2.54E-02	ko00940
**E05vsE07**			
Photosynthesis-antenna proteins	17(8.06%)	1.02579E-15	ko00196
Circadian rhythm-plant	9(4.27%)	0.001928044	ko04712
Photosynthesis	15(7.11%)	0.002216224	ko00195
**E06vsE08**			
Oxidative phoshorlation	22(24.18%)	3.15E-09	ko00190
Photosynthesis	14(15.38%)	1.86E-07	ko00195
Phenylpropanoid biosynthesis	11(12.09%)	3.99E-03	ko00940
Phenylalanine metabolism	10(10.99%)	4.63E-03	ko00360

corr_p_value represent the significance of enrichment; the value is smaller, the significance is higher. Pathway ID represent the KEGG pathway number. Based on sequence homology, 295 differentially expressed genes could be categorized into KEGG pathways.

### Expression profiles of plant hormone- related genes

**Fig 8:**
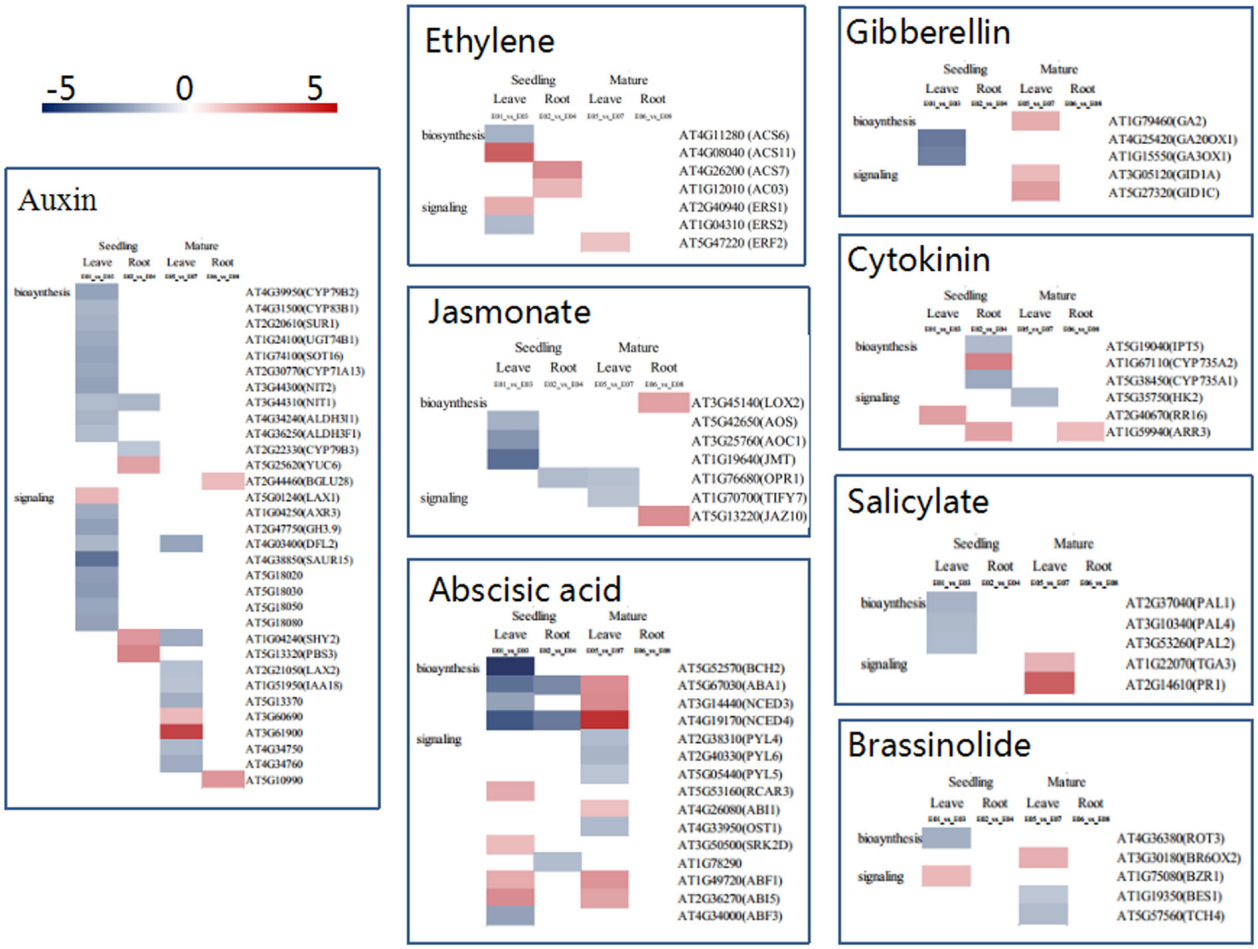
Representative gene expression profiles of hormone biosynthesis and signaling pathways in leaves and roots at seedling and mature stages. Only DEGs are shown in the heat map. Each color spot reflects the expression of a corresponding gene. Red indicates the upregulation of gene expression, blue represents the genes were downregulated, and white indicates that no signal was detected at RNA level.

We analyzed the DEGs for plant hormones and secondary metabolites, including auxin, cytokinin, gibberellin, abscisic acid (ABA), brassinosteroid, ethylene, jasmonic acid (JA) and salicylic acid (SA) (Fig 8 and S3 File). Moreover, in order to validate the accuracy of DEGs, 17 DEGs related plant hormones with differential changes in expression during different A. thaliana growth periods were selected from the DGE libraries for qRT-PCR analysis to validate the DGE data. *Actin12* (AT3G46520) was used as the reference gene for data normalization according to Begara-Morales et al[45]. The results showed that 16 genes exhibited the same changes in the direction of regulation according to both DGE and qRT-PCR, with the exception of the *AT1G16410* gene in the mature period (Table 3).

**Table 3:** Verification of DGE-seq results by qRT-PCR.

Gene	Method	E01_vs_E03	E02_vs_E04	E05_vs_E07	E06_vs_E08	Functional annotation
**AT3G12320 (ABA)**	qRT-PCR	-4.78	-3.78	0.26	NP	uncharacterized protein
	DGE profile	-5.95	-4.03	4.99	N	
**AT3G60420 (SA)**	qRT-PCR	3.53	2.26	NP	NP	phosphoglycerate mutase family protein
	DGE profile	1.94	2.65	N	N	
**AT5G45820 (IAA)**	qRT-PCR	-4.77	NP	3.47	NP	CBL- interacting serine/ threonine- protein kinase
	DGE profile	-8.15	N	7.34	N	
**AT5G37260 (ABA)**	qRT-PCR	7.51	4.14	-8.14	NP	MYB family transcription factor circadian 1
	DGE profile	6.51	4.76	-4.39	N	
**AT1G16410 (JA)**	qRT-PCR	-2.11	-0.93	NP	0.21	dihomomethionine N-hydroxylase
	DGE profile	-3.02	-4.68	N	-2.91	
**AT1G53440 (SA)**	qRT-PCR	-2.11	-0.93	NP	0.21	putative LRR receptor- like serine/ threonine- protein kinase
	DGE profile	-3.02	-4.68	N	-2.91	
**AT1G22770**	qRT-PCR	-2.67	-2.74	0.53	NP	gigantean protein
	DGE profile	-5.32	-6.36	5.27	N	
**AT1G15550 (GA)**	qRT-PCR	-1.81	NP	NP	NP	gibberellin 3-beta-dioxygenase
	DGE profile	-2.73	N	N	N	
**AT1G67110 (BR)**	qRT-PCR	NP	2.53	NP	NP	cytokinin hydroxylase
	DGE profile	N	2.42	N	N	
**AT4G28410 (ABA)**	qRT-PCR	NP	-0.88	NP	-1.04	S-alky1-thiohydroximate lyase SUR1
	DGE profile	N	-1.18	N	-1.31	
**AT4G34000 (ABA)**	qRT-PCR	-0.23	NP	NP	NP	abscisic acid responsive elements-binding factor
	DGE profile	-1.88	N	N	N	
**AT4G03400 (ABA;IAA)**	qRT-PCR	-3.07	NP	-5.14	NP	probable indole- 3- acetic acid- amido synthetase
	DGE profile	-1.41	N	-1.81	N	
**AT3G07390 (IAA)**	qRT-PCR	-2.15	NP	NP	NP	auxin-induced in root cultures protein
	DGE profile	-1.18	N	N	N	
**AT3G15450 (SA)**	qRT-PCR	3.5	NP	0.67	NP	atem-specific protein TSJT1
	DGE profile	2.09	N	1.54	N	
**AT2G38310 (ABA)**	qRT-PCR	NP	NP	-6.32	NP	abscisic acid receptor PYL4
	DGE profile	N	N	-1.27	N	
**AT1G71030(ET;GA;ABA;IAA;SA;JA)**	qRT-PCR	-1.14	-1.31	0.33	NP	putative myb family transcription factor
	DGE profile	-2.46	-3.01	3.96	N	
**AT1G78815 (CK)**	qRT-PCR	NP	1.89	NP	NP	uncharacterized protein
	DGE profile	N	1.4	N	N	

N represents that the gene was not a differentially expressed gene in this comparison. NP represents that the gene was not detected by qRT-PCR.

Specifically, the expression of ethylene biosynthesis genes *AT4G26200* (*ACS7*) and *AT1G12010* (*AC03*) was strongly increased in root compared with in leaves at seedling stage. Ethylene signaling- related genes *AT2G40940* (*ERS1*) and *AT5G47220* (*ERF2*) were up-regulated in leaves at both two growth stage. The expression of brassinosteroids, gibberellin and salicylic acid biosynthesis and signaling- related genes was induced in leaves at two growth stages. The genes of auxin and cytokinin signaling- related were up regulated in roots at two growth stages. The expression of jasmonate biosynthesis and signaling- related genes was only positively induced in roots at mature stage. ABA- related genes were positively induced in leaves at two growth stages. These findings indicated that plant hormones occur by tissue- independent when response to FZB42 volatiles.

## Discussion

In terms of plant–PGPR interactions, studies have increasingly demonstrated that bacterial volatiles play important roles in the promotion of plant growth and induced resistance. Possible mechanisms have been reported for the stimulation of growth and the elicitation of plant basal immunity by PGPR strains via VOC emissions, especially in *B*. *subtilis*. Furthermore, previous studies have shown that different response mechanisms are activated when plants are exposed to volatiles from *B*. *subtilis* and *B*. *amyloliquefaciens*. However, there has been no previous comprehensive analysis of the response mechanisms involved in *Arabidopsis*–*B*. *amyloliquefaciens* FZB42 interactions. Thus, in the present study, we used Illumina HiSeg2500 to obtain a virtually complete set of predicted genes in *Arabidopsis* regulated by the volatiles emitted from *B*. *amyloliquefaciens* FZB42 by analyzing the responses of different tissues (leaf and root) and growth stages (seedling and mature).

### Bacterial volatile regulate plant growth

Sustained exposure to *B*.*amyloliquefaciens* (FZB42) volatile emissions triggers long-term Arabidopsis plant growth promotion compared to water controls. Sustained FZB42 volatile signaling is necessary as indicated by the loss of enhanced growth when FZB42 is withdrawal early in the plant development (Figs 1 and 2). These results were consistent with the previous results[46]. At RNA level, our results demonstrated that the putative auxin efflux carrier At2g17500 was found (downregulated) at the seedling stage, which was consistent with a previous study by oligonucleotide microarray slides [21]. Although the down-regulation of the gene *At2g17500* was observed at seedling stage, it is still unclear the specific response site of plant to bacteria volatiles. However, our results showed that the gene *At2g17500* only expressed at roots. Moreover, three of the four flavonoid biosynthesis-encoding genes were downregulated in the leaves and upregulated in the roots. We first found the down-regulated of the gene *At2g17500* in roots, which putatively associated with localized auxin in the roots [21]. And a novel finding in this study is that genes encoding flavonoid biosynthesis were down regulated in leaves but up regulated in roots, which may be contributed to the activation of basipetal auxin transport. It is reported that increased auxin levels have negative effects on leaf expansion. Low auxin concentrations were favorable to leaf cell enlargement [47, 48]. Thus, FZB42 triggered the reduction of flavonoids in leaves and the accumulation of flavonoids in roots, thereby contributing to the lower ratio of auxin levels between leaves and root and then leading to increased root auxin levels and the promotion of plant growth. In addition, although it has been reported that the plant growth-promoting effects of GB03 volatiles are related to the cytokinin receptor or ethylene signaling based on *cre1* and *ein2* mutants, respectively [41], our results showed that the *cre1* and *ein2* genes were not expressed at either stage or in any tissues, which agreed with previous microarray results [42]. In contrast to the seedling stage, the flavonoid biosynthesis-encoding genes were not expressed in the roots and they were still downregulated in the leaves at the mature stage, which suggests that the response of *Arabidopsis* to volatiles produced by FZB42 differs among plant growth stages.

Genes related to cell wall modifications were examined to assess the possible regulatory control of cell expansion when exposed to FZB42 emissions. Further analysis found that genes encoding cell expansion were upregulated to allow cell expansion in the roots during the seedling and mature stages. Moreover, pectin-related genes were differentially regulated, including pectin methylesterase inhibitor, pectinase, and pectate lyase, thereby suggesting that the cell expansion regulatory mechanism is highly complex in different growth stages. In a previous microarray study where *Arabidopsis* was colonized by GB03, related cell wall modifications and cell enlargement were induced in the leaves, while transcripts associated with cell-wall loosening activity and cell expansion were upregulated [21]. These results indicate that FZB42 volatiles also induced the expression of genes involved in cell wall modifications but the response mechanism differed between these two strains.

Besides, the pathways were enriched in roots at both growth stages including unidimensional cell growth (GO:0009826), cell morphogenesis involved in differentiation (GO:0000904) and trichoblast differentiation (GO:0010054). Moreover, root hair cell differentiation (GO:0048765) and negative regulation of cytokinin mediated signaling pathway (GO:0080037) were also founded in roots at seedling stage. Most of the DEGs related to these pathways were up regulated, which indicated that a pivotal role of roots and the contribution in plant growth promotion response to FZB42 volatiles. Zhang et al. reported that GB03 volatiles promoted the plant growth by inducing greater numbers of lateral roots but not cell size or cell area. Our results are partly consistent with this previous report [21]. DEGs related to these pathways may represent FZB42 volatiles-related genes that warrant further functional study.

### Bacterial volatile regulate plant system resistance

To study the systemic plant resistance response elicited by bacterial volatiles, we assessed the possible mechanisms involved in ISR by measuring the expression levels of genes in the leaves and roots at different growth stages. Ethylene can promote specific defense responses due to environmental stresses such as wounding, flooding, and pathogen attack [49]. A previous proteome analysis identified key enzymes induced by GB03 volatiles that are involved in ethylene biosynthesis, such as aspartate aminotransferase, aspartate-semialdehyde dehydrogenase precursor, methionine adenosyltransferase 3 (MAT3), and S-adenosyl-methionine synthetase 2 (SAM-2) [12]. These four enzymes are involved in the biosynthesis of ethylene from oxaloacetate and they are required by *Arabidopsis*, where the conversion of oxaloacetate to S-adenosyl-L–methionine (SAM) is catalyzed by bacterial VOC emissions. Interestingly, in the present study, bacterial FZB42 volatiles induced the last step in ethylene biosynthesis and the signaling pathway from SAM, as shown in Fig 8 and S3 File. It is known that 1-aminocyclopropane-1-carboxylic acid (Acc) synthase (ACS) and 1-aminocyclopropane-1-carboxylate oxidase (ASO) catalyze the conversion of SAM to Acc and finally to ethylene in *Arabidopsis*, which were identified in response to VOC emissions. Similarly, the ethylene response sensor (ERS) and ethylene-responsive transcription factor (ERF) are involved in the ethylene signal transduction pathway in *Arabidopsis*, where ethylene gas is perceived by ERS and ERF to provide enhanced disease resistance when they are overexpressed [50]. However, as shown in Fig 8 and S3 File, the DEGs related to ethylene biosynthesis and signaling differed among tissues and growth stages, which indicates that they may be both tissue- and growth stage-dependent. In conclusion, our analysis suggests that VOC-mediated ISR elicitation is not a common mechanism among all PGPR in the rhizosphere.

A previous microarray analysis detected the upregulation of several antioxidant genes at 72 h after volatile emission [21]. In addition, a study based on proteome data suggested that bacterial volatile emissions upregulated the levels of antioxidant enzymes, thereby indicating a potential role for ROS-scavenging enzymes [12]. In the present study, our DEGs data suggested that the genes for some ROS-scavenging enzymes were induced to different degrees by FZB42 VOC emissions. Volatiles released from GB03 triggered growth promotion and ISR via cytokinin and ethylene signaling pathways, whereas the volatiles produced by IN937a appeared to operate through cytokinin- and ethylene-independent pathways. These findings are difficult to explain given their similar volatile profiles [4, 5], thereby implicating the presence of other undetected or identified volatiles, which may operate via different signaling pathways in plants. We found that the same volatiles produced by FZB42 were also emitted by GB03 and IN937a. Interestingly, some potential volatiles related to resistance against fungi and nematodes were detected in strain FZB42 but not in GBO3 or IN937a (data not shown). Moreover, our present results showed that the two pathways (regulation of plant-type hypersensitive response (GO:0010363) and killing of cells of other organism (GO:0031640)) were strongly induced in leaves and the other pathway defense response to nematode (GO:0002215) with up- regulated genes was significantly enriched in roots at both growth stages. These results and previous analyses of the variation in volatile profiles among PGPR strains suggest that diverse VOC metabolic mechanisms exist, which supports the idea that volatiles could serve as taxonomic markers for PGPR, similar to those used in other microbial systems [51]. Thus, the response mechanisms differ in plants to the diverse volatiles emitted by GB03, IN937a and FZB42.

### Bacteria volatiles induce plant system tolerance

Volatiles may trigger plant tolerance of abiotic stresses, such as salt stress, drought stress and/or nutrient deficiencies. It is known that ROS are related to abiotic stresses such as high salt and drought, as well as being induced to resist pathogens [52]. Recently, some studies have shown that PGPR may confer plants with resistance to abiotic stresses such as salt and drought, which is referred to as IST. Thus, our above results and those of previous studies indicate that bacterial volatiles play important roles in modulating the damage levels attributable to ROS.

*Arabidopsis* plants exposed to GB03 volatiles had greater salt tolerance compared with the control plants due to the lower accumulation of Na^+^ in both the shoots and roots [53]. In particular, the GB03 volatiles concurrently downregulated and upregulated *AtHKT1* expression in the roots and shoots, respectively [54]. It was reported that the sodium transporter (HKT)1 may mediate VOC-induced salt tolerance. The presence of *AtHKT1* in the shoots excludes Na^+^ from the leaves and restricts the uploading of Na^+^ to the aerial portions of plants from the roots [55, 56]. In addition, it has been shown that *AtHKT1* facilitates the shoot-to-root recirculation of Na^+^ [57]. The plant treated with GB03 volatiles in root-to-shoot ratio of the Na^+^ levels is higher than control plants, thereby supporting the role of *AtHKT1* in controlling Na^+^ in the roots [54]. Moreover, it can lead to greater Na^+^ level in the roots but less accumulation of Na^+^ in the shoots in GB03 VOC-treated plants [54]. We found that the FZB42 VOC-treated *Arabidopsis* plants exhibited down regulated *HKT1* expression in the roots at both the seedling and mature stages. However, *HKT1* expression was not detected in the leaves, irrespective of the plant growth stages. Our results and those obtained in previous studies suggest that FZB42 VOC regulates the expression of *HKT1*, which may explain VOC-induced salt tolerance to some extent.

In addition, bacterial volatiles can help plants withstand drought. Thus, GB03 and another rhizobacterium, *P*. *chlororaphis* O6, were reported to increase drought tolerance via volatiles in *Arabidopsis* [58, 59]. It was also shown that volatiles increased drought tolerance in a manner that was independent of the accumulation of ABA [59]. As mentioned above, the genes involved in the ABA signal transduction pathway were differentially regulated in different stages and tissues, thereby suggesting that the ABA synthesis and signaling pathways respond to FZB42 volatiles. Moreover, Cho et al. reported that the bacterial “scent” or 2,3-butanediol was involved in the accumulation of SA under drought stress [58]. A previous proteomic analysis demonstrated that GB03 volatiles induced ethylene as well as JA and SA signaling pathways in *Arabidopsis*. JA response (*VSP1* and *PDF1*.*2*) and SA response genes (*PR1* and *FMO1*) were upregulated either in the leaves or roots and at the seedling or mature stages, which agreed with the results of the proteomic analysis [12]. These results suggest that bacterial volatiles regulated plant by inducing ethylene, JA, and SA signaling. However, we found that the other genes involved in JA and SA biosynthesis and signaling were differentially regulated by volatiles in *Arabidopsis*. Many previous studies have shown that SA, ethylene, and JA signaling are complex and antagonistic [60]. Another study showed that ISR-induced ET/JA signaling and SAR-induced SA signaling may have synergistic effects. Thus, further experiments are needed to examine the possible regulation of the response and the hormone levels after treatment with bacterial volatiles.

### The response pathways of plant revealed the tissue-specific and/ or growth stage-specific

KEGG pathway analysis showed that glucosinolate biosynthesis was correlated with samples from leaves at the seedling stage, but limonene and pinene degradation, and/or stilbenoid, diarylheptanoid, and gingerol biosynthesis were only correlated with root samples treated with FZB42 at the seedling stage. This indicates that the responses to VOC differed among tissues. Mature stage-specificity was observed for the oxidative phosphorylation pathway in roots. We found that 12 downregulated genes (including *CYP79F1*, *CYP83A1*, *CYP83B1*, *CYP79B2*, *SUR1*, and *UGT74B1*) involved in glucosinolate biosynthesis were expressed in contrast to the water control treatment. CYP450 genes are involved with secondary metabolites that help plants to resist various stressful conditions [61]. Indole derivatives are initially derived from the tryptophan- dependent pathway by cytochrome P450, CYP79B3, and CYP79B2 [62–64]. Furthermore, previous studies have shown that genes involved with indole glucosinolate production are significantly upregulated when treated with bacterial pathogens [65]. Interestingly, our results differed from those obtained previously, which may be attributable to differences in the strain employed or the plant growth stages.

In addition to the tissue-specific response pathways at different growth stages, we found that two KEGG pathways, i.e., phenylpropanoid biosynthesis and phenylalanine metabolism, had significant correlations with roots, where these correlations were independent of the growth stage. Our results showed that most of the genes in these two pathways were upregulated compared with the control. Previously, a role has been suggested for the phenylpropanoid pathway in plant resistance to pathogen attack [66, 67], where the production of monolignols strengthens the lignification of cell walls and provides mechanical resistance against pathogens [68]. In addition to providing a physical barrier, flavonoids are metabolic products from the phenylpropanoid pathway with important roles in resistance [67, 69].

However, to our surprise, photosynthesis had a significant correlation with the growth stage, where this correlation was independent of the plant tissue type. Excluding the *AT2G40100* gene, all of the genes in this pathway were downregulated at the seedling stage whereas all of the genes were upregulated at the mature stage. Most of the genes were involved with photosystem II light harvesting complex protein, which is consistent with previous results [70]. In 2008, Zhang et al. reported that the photosynthetic efficiency was increased by the presence of a PGPR, *B*. *subtilis* GB03 [53]. Moreover, the chlorophyll content was increased by the same strain GB03, although some enzymes in this pathway were also downregulated whereas other enzymes were upregulated according to the results obtained by proteome analysis [12]. By examining total chlorophyll (Chl. A+b) content (Fig 3), our data also showed FZB42 volatiles increases chlorophyll content compared with water as control. Thus, it is possible that the decreased activity of photosynthesis genes detected in the present study only improves the fitness of plants in the seedling stage when resisting pathogens. By contrast, the increased activity of photosynthesis genes may play a key role in the mature stage by increasing carbon gain and/or seed production in the host plant.

However, there are other cases to consider. Previously, it was found that the Volatiles released by GB03 upregulated the genes for Fe-deficiency-induced transcription factor 1 (*FIT1*), ferric reductase *FRO2*, and iron transporter *IRT1* [71]. Iron is indispensable for the photosynthetic apparatus. Thus, the genes mentioned above should be upregulated when photosynthesis is more active in the mature stage, but our results showed that FZB42 Volatiles decreased the mRNA levels of *AT2G28160* (*FIT1*), *AT1G01580* (*FRO2*), and *AT4G19690* (*IRT1*) in the roots at the mature stage. This may indicate that strain-specific networks exist within the roots or in different plant growth stages. A few studies have addressed the relationship between photosynthetic regulation and PGPRs [70]. In particular, Zhang et al. reported that GB03 enhanced the photosynthetic activity by reducing the ABA levels [53]. Our results showed that the ABA levels decreased during the seedling stage when the genes related to photosynthesis were downregulated. Interestingly, the ABA levels increased in the leaves at the mature stage when there was an increase in photosynthesis according to the gene expression levels.

## Conclusion

**Fig 9:**
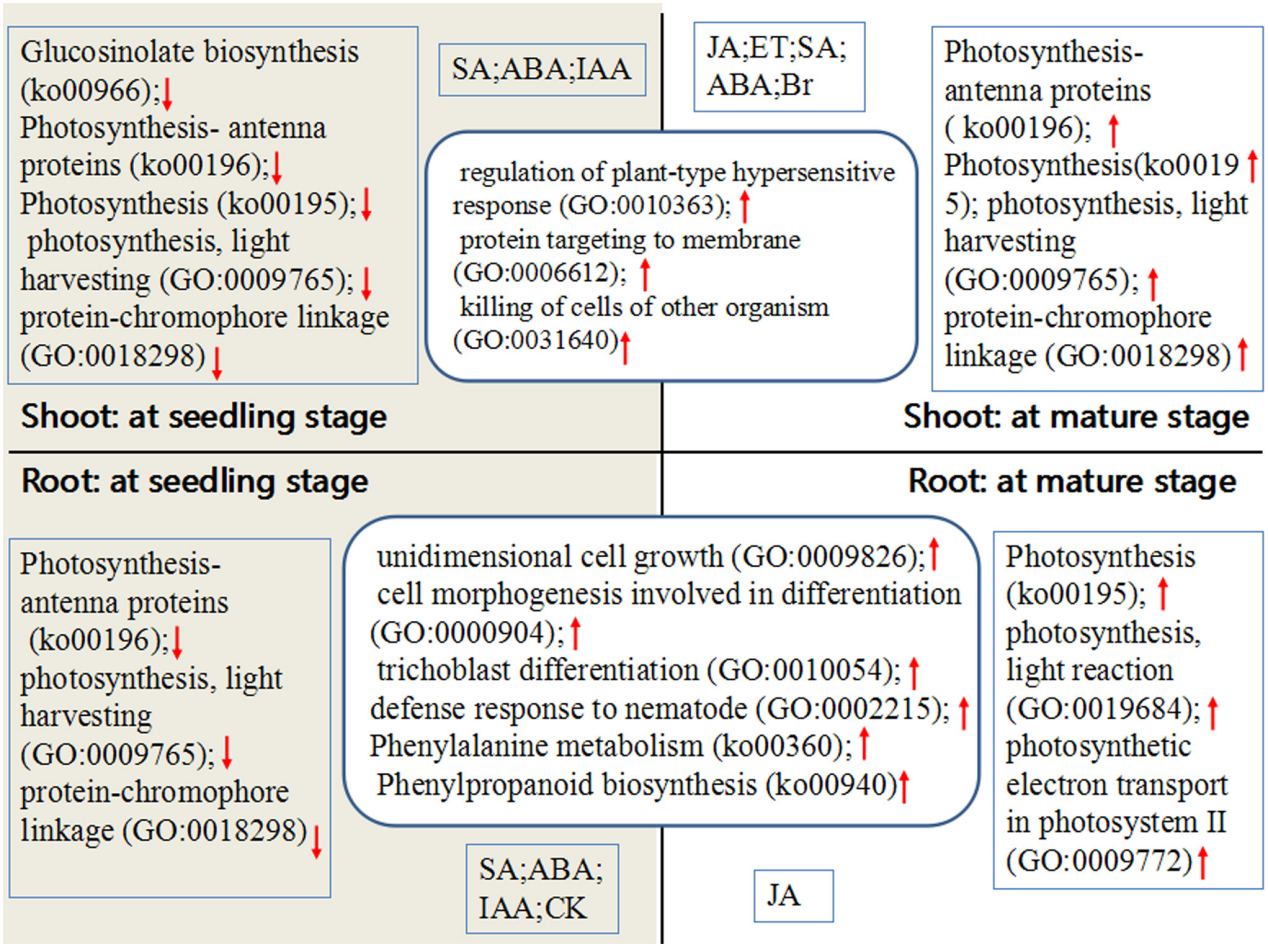
A putative model for the response mechanism of plant to FZB42 volatiles at different tissues and growth stages. The arrow up or down represent the up or down of genes related to GO and KEGG pathways.

In this study, we obtained high throughput sequencing data on the plant response to FZB42 volatiles. Although we think that the response mechanism of plant to bacteria volatiles was a complex process, we here picture a sample model for volatiles-response mechanism according to our data. As shown in Fig 9, we think that volatiles may induce the plant sustained resistance and growth at seedling and mature stages. In leaves, FZB42 volatiles mainly induced the plant resistance whatever which growth stage. And also FZB42 trigger the expression of DEGs related to the growth and resistance at roots whatever growth stage. Moreover, in roots, we found DEGs related to defense nematode at seedling and mature stages. DEGs related to plant hormones were enriched at different tissues and growth stages. In addition, we found some DEGs enriched were not independent- tissues and/ or growth stages (Glucosinolate biosynthesis (ko00966)). Some DEGs related to photosynthesis were down regulated at seedling stage and up regulated at mature stage, which may exist other regulated means when response to FZB42 volatiles. In conclusion, FZB42 volatiles can induce plant growth and resistance once response to bacteria volatiles.

## Supporting Information

S1 File (XLS)

S2 File (XLSX)

S3 File (XLSX)

S1 Table The details of the primers used for real-time PCR in the experiment. (DOC)

S2 Table Numbers of differentially expressed genes (DEGs) in each comparison. (DOC)
