# Supplementary material for: Comparative Digital Gene Expression Analysis of the Arabidopsis Response to Volatiles Emitted by Bacillus amyloliquefaciens
Source: PLoS One. 2016 Aug 11;11(8):e0158621. doi: 10.1371/journal.pone.0158621 (PMC4981348; doi:10.1371/journal.pone.0158621)
Supplement: S1 Table — (DOC) [file pone.0158621.s004.doc]

**Supplementary Table 1.The details of the primers used for real-time PCR in the experiment.**

|  | **Sequence ID** | **Primer sequence (5′–3′)** |
| --- | --- | --- |
| 1 | AT3G12320-F | TGGTGCTTCTTCACTTCACG |
|  | AT3G12320-R | GAACATCTCCATCATCCTCTGG |
| 2 | AT3G60420-F | GATACCGCACCCAATCTTTATT |
|  | AT3G60420-R | CCCATTCTGGCACCTCTTTA |
| 3 | AT5G45820-F | CTGAAGTGATAGGCAAGAAAGGTT |
|  | AT5G45820-R | TCGTGAAACGGAAGAAATCC |
| 4 | AT5G37260-F | CTGAAGATGGCAAGAAGAAGC |
|  | AT5G37260-R | TTTGAACTCCGACACTACCACT |
| 5 | AT1G16410-F | CGTGCCATCACCATAAACTC |
|  | AT1G16410-R | TTGTCTCCGATTGTCTCCAT |
| 6 | AT1G53440-F | CGCACACCATTCTTCCTTGT |
|  | AT1G53440-R | CACCGTCATTGCCTAACCA |
| 7 | AT1G22770-F | GGAGCTGTCTTTCTCCGTTG |
|  | AT1G22770-R | CATAGCGCATCAACAACCTG |
| 8 | AT1G15550-F | GTCTCTTCGGGCTACCTGTC |
|  | AT1G15550-R | GTGAAACCTTCGGACCACAT |
| 9 | AT1G67110-F | ACTGCTCTTCCATCCACCAC |
|  | AT1G67110-R | TCATCTCGGTCTCCGTTAGG |
| 10 | AT4G28410-F | CGATGAACGCAAGACAACAG |
|  | AT4G28410-R | TTTGGAAGCAAGGGTAGACG |
| 11 | AT4G34000-F | CAACAAACGCTGGGAGAGAT |
|  | AT4G34000-R | CCATAGAATCCGCCATTGA |
| 12 | AT4G03400-F | TGTTCCGTTTACTCGCCATAG |
|  | AT4G03400-R | CCTCCCTCCTTCCCTTATTG |
| 13 | AT3G07390-F | CCTCTTATCAAACACCAAACCAC |
|  | AT3G07390-R | ATTAGCGAGACGAAACAAGCA |
| 14 | AT3G15450-F | GCGAAACTCTCTCCGACTTC |
|  | AT3G15450-R | CCACAGAACAACCTTTGACG |
| 15 | AT2G38310-F | GTCCTTCTTCCGCCGTATC |
|  | AT2G38310-R | ACCAACCTCGTGTGTGTGAA |
| 16 | AT1G71030-F | AAGATTGCCAGGACGAACC |
|  | AT1G71030-R | GTGTGATGGTGGAGACGATG |
| 17 | AT1G78815-F | AAAGGCAAAGGCATAGCAGA |
|  | AT1G78815-R | CGTGTTCCAGTCTCGTCGT |
