# Supplementary material for: Comparative Digital Gene Expression Analysis of the Arabidopsis Response to Volatiles Emitted by Bacillus amyloliquefaciens
Source: PLoS One. 2016 Aug 11;11(8):e0158621. doi: 10.1371/journal.pone.0158621 (PMC4981348; doi:10.1371/journal.pone.0158621)
Supplement: S2 Table — (DOC) [file pone.0158621.s005.doc]

**Supplementary Table 2.**

**Numbers of differentially expressed genes (DEGs) in each comparison**

| DEG Set | ALL DEGs | Upregulated | Downregulated |
| --- | --- | --- | --- |
| E01_vs_E03 | 1,507 | 615 | 892 |
| E02_vs_E04 | 820 | 469 | 351 |
| E05_vs_E07 | 1512 | 867 | 645 |
| E06_vs_E08 | 367 | 266 | 101 |
